# Additive-controlled chemoselective inter-/intramolecular hydroamination via electrochemical PCET process

**DOI:** 10.3762/bjoc.20.27

**Published:** 2024-02-12

**Authors:** Kazuhiro Okamoto, Naoki Shida, Mahito Atobe

**Affiliations:** 1 Graduate School of Engineering, Yokohama National University, 79-7 Tokiwadai, Hodogaya-ku, Yokohama, Kanagawa 240-8501, Japan

**Keywords:** amidyl radical, cyclic voltammetry, electrosynthesis, hydroamination, proton coupled electron transfer

## Abstract

Electrochemically generated amidyl radical species produced distinct inter- or intramolecular hydroamination reaction products via a proton-coupled electron transfer (PCET) mechanism. Cyclic voltammetry (CV) analysis indicated that the chemoselectivity was derived from the size of the hydrogen bond complex, which consisted of the carbamate substrate and phosphate base, and could be controlled using 1,1,1,3,3,3-hexafluoro-2-propanol (HFIP) as an additive. These results provide fundamental insights for the design of PCET-based redox reaction systems under electrochemical conditions.

## Introduction

**Figure 1:**
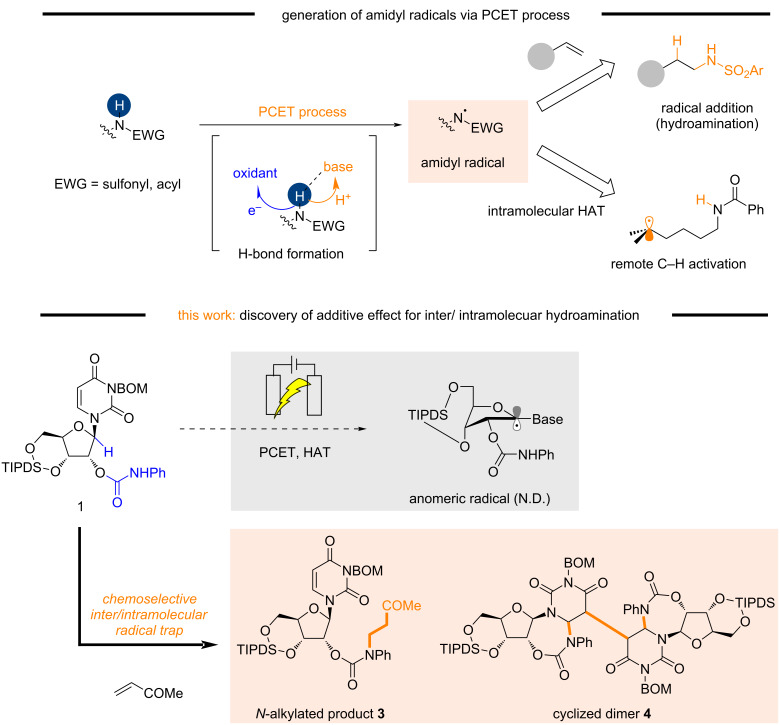
Application of amidyl radical species generated by PCET.

Proton-coupled electron transfer (PCET) enables the generation of various radical species under ambient conditions (Figure 1, top) [1]. In PCET processes, hydrogen bond formation between weak bases and acidic X–H bonds (X = N, O, C) is a key step, which is followed by concerted proton- and electron-transfer to give the corresponding radical species through oxidative X–H bond cleavage. One such species is the amidyl radical, which is broadly synthetically useful as a nitrogen source in hydroamination reactions and as a hydrogen atom transfer (HAT) reagent for remote C–H activation [2–8]. Recent advances in photoredox and electrochemical PCET reactions have significantly expanded the substrate scope of amidyl-radical-based molecular transformations because the harsh acidic and high-temperature conditions required in the classical Hofmann–Löffler–Freytag reaction can be avoided [9].

The initial aim of this study was the electrochemical generation of an amidyl radical as a HAT source for the synthesis of 1’-*C* functionalized nucleosides via the generation of an anomeric radical species from uridine derivative **1** (Figure 1, bottom) [10]. Although the HAT reaction failed, remarkable inter- and intramolecular chemoselectivities were observed in the hydroamination reaction. We investigated this phenomenon and found that complete inter-/intramolecular chemoselectivity could be achieved by modifying the reaction conditions, despite the presence of both inter- and intramolecular radical acceptor moieties. Therefore, we investigated the origin of this selectivity under electrochemical conditions.

## Results and Discussion

Anodic oxidation of uridine derivative **1** was performed in a CH_2_Cl_2_/Bu_4_NPF_6_ (0.1 M) electrolyte system using a carbon felt (CF) anode and a Pt cathode in the presence of methyl vinyl ketone (MVK) as a radical acceptor (Table 1). Tetrabutylammonium dibutyl phosphate (phosphate base), which operates as a PCET initiator through hydrogen bond formation with the N–H bond of amide/carbamate [11], was used as an additive. As a result, *N*-alkylated product **3** was exclusively obtained, implying that the expected HAT at the 1’-*C* position to afford **2** (Table 1, entry 1) had not occurred. In contrast, the reaction efficiency was significantly decreased in the absence of the phosphate base (Table 1, entry 2), and electricity is necessary to proceed the reaction (Table 1, entry 3); thus, the phosphate base plays a crucial role in *N*-alkylation, while its basicity is insufficient to promote aza-Michael addition (p*K*_a_ of the conjugate acid of the phosphate base is 1.72 in H_2_O) [12]. Furthermore, *N*-alkylation proceeded in a divided cell (anodic chamber); thus, the possibility of conjugate addition of a cathodically generated carbamate anion was ruled out, prompting us to consider that *N*-alkylation proceeded via a radical mechanism. On the other hand, the addition of 1,1,1,3,3,3-hexafluoro-2-propanol (HFIP) led to the predominant formation of cyclized dimer **4** without *N*-alkylation, whereas the use of AcOH provided *N*-alkylated product **3** (Table 1, entries 5 and 6). When acetonitrile (MeCN) was used as the solvent, cyclized dimer **4** was obtained (Table 1, entry 7).

**Table 1:** Electrochemical oxidation of **1** under varying conditions.

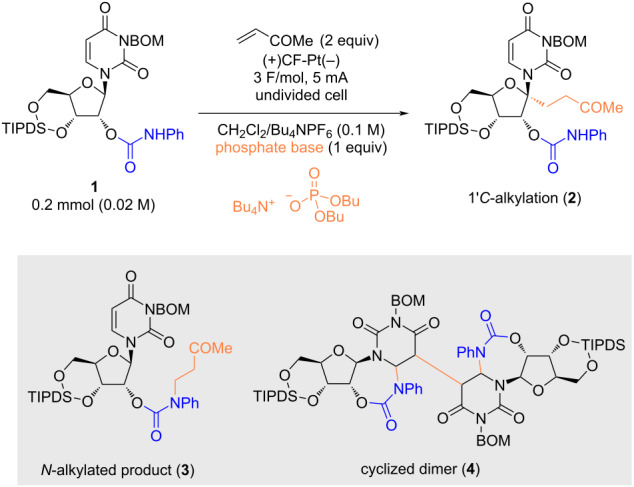			

Entry	Deviation from standard conditions	Yield [%]^a^	Recovered **1** [%]^a^

1	none	57, 49^b^ (**3**)	17
2	without phosphate base	13 (**3**)	76
3	without electricity	N.R.	92
4	divided cell (anodic chamber)	41 (**3**)	27
5	HFIP (2 equiv) as an additive	42, 27^b^ (**4**)	32
6	AcOH (2 equiv) as an additive	10 (**3**)	51
7	MeCN instead of CH_2_Cl_2_	17 (**4**)	28

^a^Yield was determined based on ^1^H NMR by using benzaldehyde as an internal standard, and recovery rate of **1** was determined by the integral of H-1’ proton. ^b^Isolated yield.

**Figure 2:**
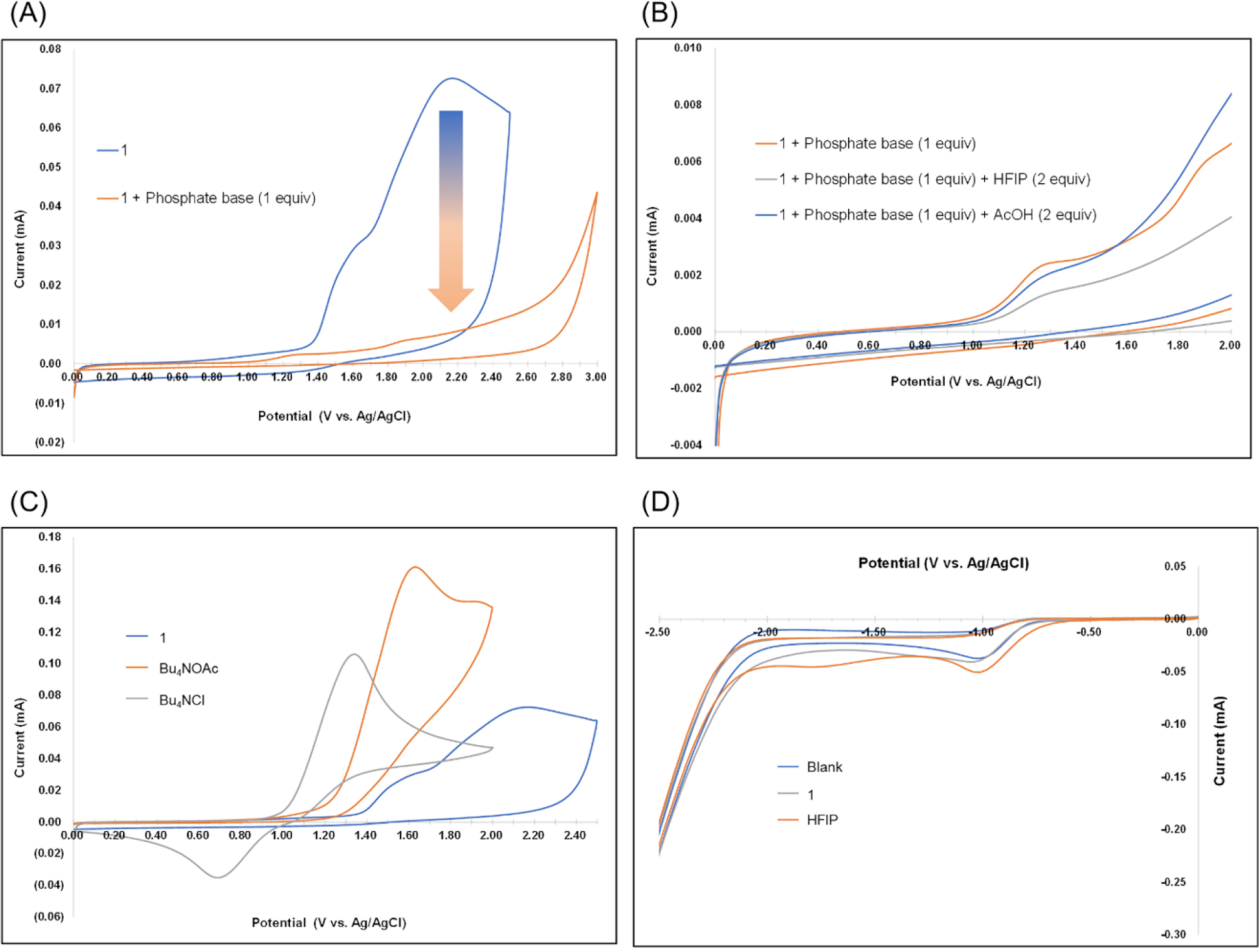
(A) Effect of phosphate base on the cyclic voltammogram of **1**. (B) Cyclic voltammograms of **1** in the presence of additives (AcOH or HFIP). (C) Comparison of oxidation potentials of **1** using Bu_4_NOAc or Bu_4_NCl. (D) Cyclic voltammograms for the cathodic side. All cyclic voltammograms were recorded in CH_2_Cl_2_/Bu_4_NPF_6_ (0.1 M). Sample concentration was 0.01 M. A glassy carbon anode (φ 3 mm) and Pt cathode (φ 3 mm) were used. Scan rate = 100 mV/s.

Next, **1** was subjected to cyclic voltammetry (CV) measurements under varying conditions (Figure 2). An oxidation wave was observed at approximately +1.4 V (Figure 2A). The oxidation current of this wave decreased significantly in the presence of a phosphate base and the subsequent addition of HFIP enhanced this phenomenon (Figure 2B, grey line). In contrast, using AcOH instead of HFIP did not affect the oxidation current (Figure 2B, blue line). We considered that the inter- and intramolecular chemoselectivities were derived from the p*K*_a_ of the proton sources.

The pre-organization of the amide substrate and phosphate bases is an important process in PCET [13]. Recently, Gschwind et al. published a detailed NMR spectroscopic analysis of a PCET-mediated hydroamination reaction, which indicated that the p*K*_a_ of the proton source (PhSH or PhOH in the study) determines the size of the hydrogen bond complex. PhSH as the more acidic additive (p*K*_a_ = 6.62 in H_2_O) provided better results in the PCET-induced intramolecular hydroamination reaction compared to the less acidic PhOH (p*K*_a_ = 9.95 in H_2_O) because PhSH supplied free protons (H^+^) and contributed to the persistence of small aggregates composed of the amide and phosphate base [14]. On the other hand, owing to the insufficient dissociation constant between the proton and phenoxide in PhOH, the PhOH molecule is included in the hydrogen bond network along with the tetrabutylammonium cation (Bu_4_N^+^) to form a large aggregate. The hydrogen bonding between the amide and phosphate base in the small aggregates was stronger than in the large aggregates, which significantly enhanced amidyl radical generation through the PCET mechanism.

**Figure 3:**
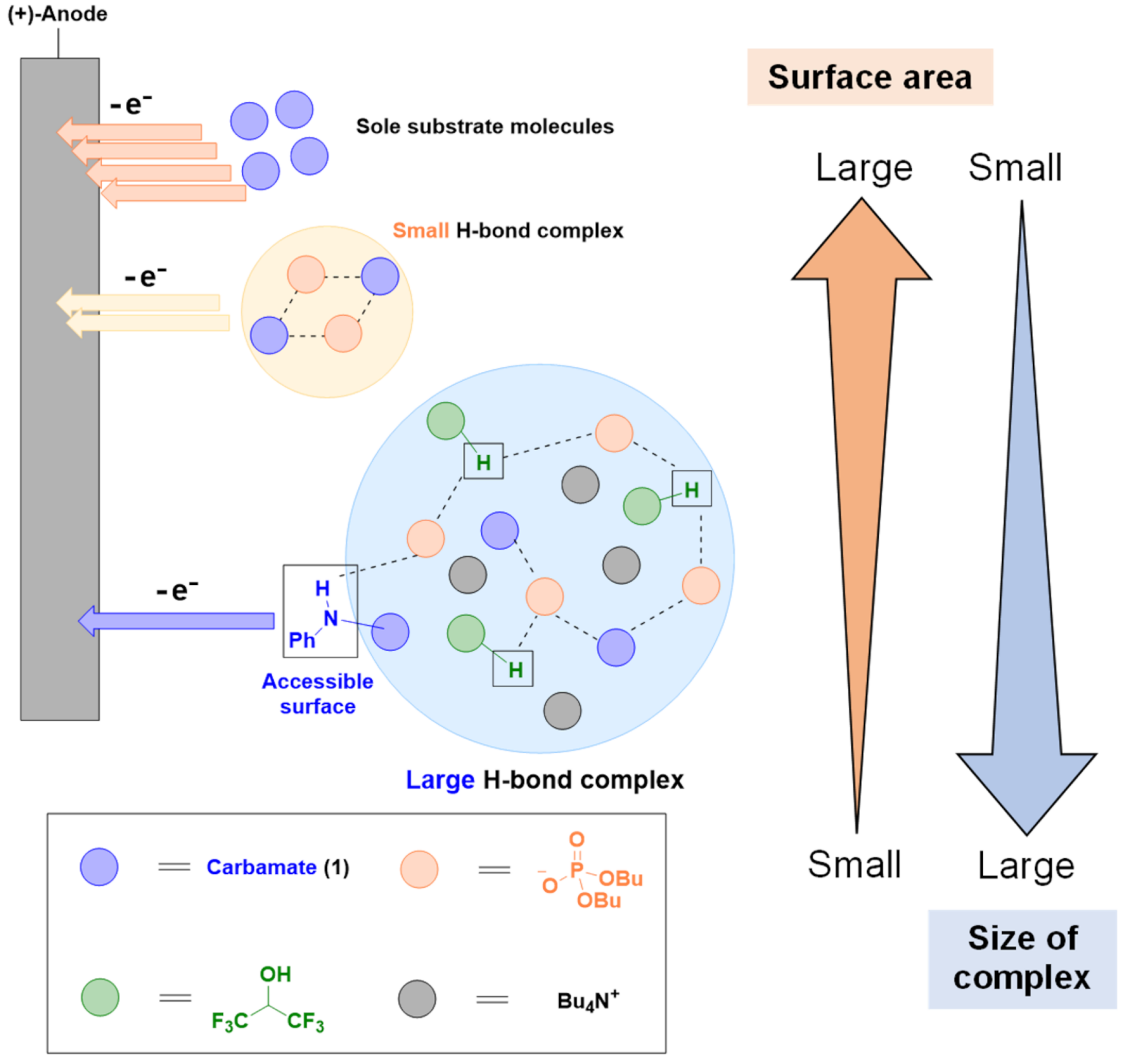
Plausible models illustrating the size effect of the hydrogen bond complex on the interaction efficiency with electrode surfaces.

The above studies provided us with valuable insights into the intriguing electrochemical behavior of **1** (Figure 3). Hydrogen bond formation between **1** and the phosphate base yielded small aggregates, the interaction efficiency of which with the electrode surface was lower than that of **1** because the relatively large hydrodynamic radius of the aggregates decreased the number of electrode-accessible molecules. This increase in the hydrodynamic radius resulted in a decrease in the oxidation current. In the present study, HFIP (p*K*_a_ = 9.30 in H_2_O) [15] is less acidic than AcOH (p*K*_a_ = 4.76 in H_2_O) with a p*K*_a_ value similar to that of PhOH, which forms large aggregates under PCET conditions, as described above. Therefore, analogously, HFIP is expected to be included in the hydrogen-bonded complex. The resulting large aggregates further impeded access to the electrode surface, and a further decrease in the oxidation current was observed in the presence of HFIP (Figure 2B, grey line). In contrast, the more acidic AcOH supplied free protons, which enabled the persistence of small aggregates; thus, the current was not affected by the presence of AcOH (Figure 2B, blue line). However, in the presence of AcOH, the *N*-alkylation yield was low (Table 1, entry 6) owing to the competitive Kolbe oxidation of the cathodically generated acetate anion. In fact, the oxidation potential of Bu_4_NOAc is lower than that of **1** (Figure 2C, orange line).

A decrease in the oxidation current can be considered as a decrease in the diffusion coefficient of the hydrogen bond complex; thus, we attempted to reproduce the CV pattern by computational simulation (Figures S1 and S2 in Supporting Information File 1) [16]. The results indicated that an excessively small diffusion coefficient (1/10- or 1/100-fold) is required to reproduce a CV pattern similar to that observed experimentally. Because the reported diffusion coefficient is only twice as small as that of the sole amide molecule [14], this simulated value is unrealistic, and we assumed that the diffusion coefficient did not affect the oxidation current.

**Figure 4:**
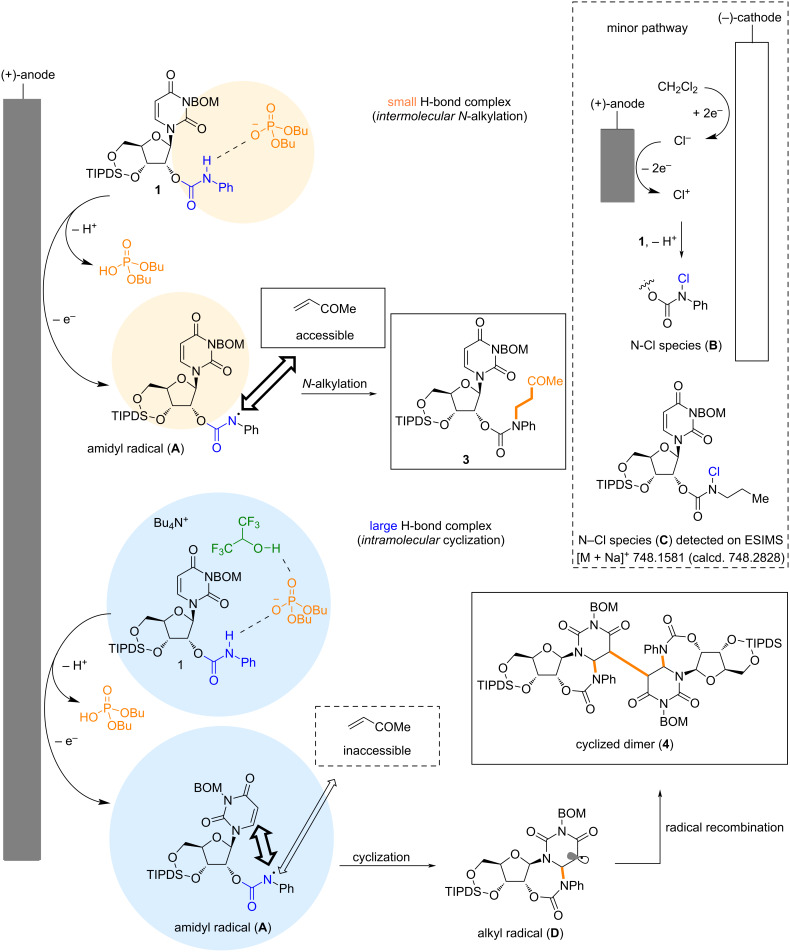
Plausible mechanism for the inter-/intramolecular hydroamination of **1**.

In cathodic events, the reduction of CH_2_Cl_2_ primally occurred under standard conditions because the reduction wave of the blank solution appeared at approximately −1.0 V (Figure 2D, blue line). The resulting cathodically generated chloride ion (Cl^−^) has a lower oxidation potential than **1** (Figure 2C, grey line); thus, it was subsequently oxidized on the anode to afford the halonium ion (Cl^+^), which can react with **1** to form unstable N−Cl species (**B**) in situ (Figure 4). Although we cannot detect the chlorinated intermediate of **1**, electrolysis of *N*-propylcarbamate derivative under standard conditions gave the corresponding N−Cl species (**C**) as an unstable compound. We considered that this result as direct evidence for the plausibility of the existence of N−Cl species which driving the minor reaction pathway.

Further single-electron reduction affords the amidyl radical [17], which can react with MVK. Because *N*-alkylation also proceeded in the absence of a phosphate base but in a low yield (Table 1, entry 2), it can be concluded that only the N−Cl species contributed to *N*-alkylation in this case.

Based on the experimental and simulation results, we propose a plausible mechanism for the inter- and intramolecular hydroamination of **1** (Figure 4). In the *N*-alkylation reaction, anodic oxidation of a small hydrogen-bonded complex produces amidyl radical **A**. The hydrophobic MVK molecule was excluded from the highly polar environment of this complex, but the resulting amidyl radical could access MVK because it still had a large surface area for interaction with the solution interface. As mentioned above, the amidyl radical can also be generated through N−Cl species **B**.

However, the large hydrogen-bond complex, which included HFIP, prevented amidyl radical access to MVK. In this case, intramolecular radical trapping by the uracil nucleobase was preferred, leading to the formation of the cyclized alkyl radical **D**. Continuous radical recombination furnished dimer **4**.

## Conclusion

We observed additive-controlled inter- and intramolecular chemoselectivity in the hydroamination of **1**. Detailed CV analysis indicated that the size of the hydrogen bond complex determined the selectivity, and HFIP played a crucial role in expanding the hydrogen bond network. These results provide fundamental insights beneficial for the design of PCET-based redox reaction systems under electrochemical conditions.

## Experimental

### General procedure of anodic oxidation

Compound **1** (145 mg, 0.2 mmol), Bu_4_NPF_6_ (387 mg, 1 mmol), CH_2_Cl_2_ (10 mL), phosphate base (90 mg, 0.2 mmol) and methyl vinyl ketone (32.7 μL, 0.4 mmol) were added to a test tube, which was then subjected to a constant electrical current of 5 mA (3 F/mol, 57.9 C) through the CF anode (1 × 1 cm) and the Pt cathode (1 × 1 cm). The reaction mixture was concentrated in vacuo and Et_2_O (20 mL) was added. The resulting precipitate was removed by filtration through a short silica gel pad under reduced pressure. The filtrate was concentrated in vacuo and the resulting residue was subjected to ^1^H NMR spectroscopy or column chromatography. A divided-cell experiment was performed using an H-type cell (4G glass filter). Compound **1** (0.2 mmol), Bu_4_NPF_6_ (387 mg, 1 mmol), phosphate base (90 mg, 0.2 mmol), CH_2_Cl_2_ (10 mL), and methyl vinyl ketone (32.7 μL, 0.4 mmol) were added to the anode chamber, and CH_2_Cl_2_ (10 mL), and Bu_4_NPF_6_ (387 mg, 1 mmol) were added to the cathode chamber. The anolyte was transferred to a round-bottomed flask, and the solvent was removed in vacuo. Et_2_O (20 mL) was added to the crude mixture, and the resulting precipitate was removed by filtration through a short silica gel pad under reduced pressure. The filtrate was concentrated in vacuo and the resulting residue was subjected to ^1^H NMR spectroscopy or column chromatography.

## Supporting Information

File 1 Detailed experimental procedures, CV simulation, copies of NMR spectra.

## Data Availability

All data that supports the findings of this study is available in the published article and/or the supporting information to this article.

## References

[1] Murray P R D, Cox J H, Chiappini N D, Roos C B, McLoughlin E A, Hejna B G, Nguyen S T, Ripberger H H, Ganley J M, Tsui E (2022). Chem Rev.

[2] Xiong P, Xu H-C (2019). Acc Chem Res.

[3] Fazekas T J, Alty J W, Neidhart E K, Miller A S, Leibfarth F A, Alexanian E J (2022). Science.

[4] Wang F, Stahl S S (2019). Angew Chem, Int Ed.

[5] Zhu Q, Graff D E, Knowles R R (2018). J Am Chem Soc.

[6] Davies J, Svejstrup T D, Fernandez Reina D, Sheikh N S, Leonori D (2016). J Am Chem Soc.

[7] Choi G J, Zhu Q, Miller D C, Gu C J, Knowles R R (2016). Nature.

[8] Xu F, Zhu L, Zhu S, Yan X, Xu H-C (2014). Chem – Eur J.

[9] Hu X, Zhang G, Bu F, Nie L, Lei A (2018). ACS Catal.

[10] Gimisis T, Chatgilialoglu C (1996). J Org Chem.

[11] Gentry E C, Knowles R R (2016). Acc Chem Res.

[12] Kumler W D, Eiler J J (1943). J Am Chem Soc.

[13] Darcy J W, Koronkiewicz B, Parada G A, Mayer J M (2018). Acc Chem Res.

[14] Berg N, Bergwinkl S, Nuernberger P, Horinek D, Gschwind R M (2021). J Am Chem Soc.

[15] Dyatkin B L, Mochalina E P, Knunyants I L (1965). Tetrahedron.

[16] Izumiya R, Atobe M, Shida N (2023). Electrochemistry.

[17] Kim H, Kim T, Lee D G, Roh S W, Lee C (2014). Chem Commun.

