# Supplementary material for: Additive-controlled chemoselective inter-/intramolecular hydroamination via electrochemical PCET process
Source: Beilstein J Org Chem. 2024 Feb 12;20:264–71. doi: 10.3762/bjoc.20.27 (PMC10877074; doi:10.3762/bjoc.20.27)
Supplement: File 1 — Detailed experimental procedures, CV simulation, copies of NMR spectra. [file Beilstein_J_Org_Chem-20-264-s001.pdf]

BEILSTEIN JOURNAL OF ORGANIC CHEMISTRY

Supporting Information

for

Additive-controlled chemoselective inter-/intramolecular hydroamination via electrochemical PCET process

Kazuhiro Okamoto, Naoki Shida and Mahito Atobe

Beilstein J. Org. Chem. **2024,** *20,* 264–271. doi:10.3762/bjoc.20.27

Detailed experimental procedures, CV simulation, copies of NMR spectra

License and Terms: This is a supporting information file under the terms of the Creative Commons Attribution License (https://creativecommons.org/licenses/by/4.0). Please note that the reuse, redistribution and reproduction in particular requires that the author(s) and source are credited and that individual graphics may be subject to special legal provisions.
The license is subject to the *Beilstein Journal of Organic Chemistry* terms and conditions: (https://www.beilstein-journals.org/bjoc/terms)

Table of contents

**I**	Experimental section	S2
**II**	Simulation for cyclic voltammogram (Figures S1 and S2)	S8
**III**	^{1}H and ^{13}C NMR spectra of compounds	S10

I. Experimental section

General information

All reactions were performed under an argon atmosphere, unless otherwise noted. ^{1}H and ^{13}C NMR spectra were recorded in $CDCl_3$ using a Bruker DRX500 spectrometer (^{1}H 500 MHz, ^{13}C 126 MHz). Tetramethylsilane (^{1}H, 0.00 ppm) and $CHCl_3$ (^{1}H, 7.26 ppm, ^{13}C, 77.16 ppm) were used as internal standards. Mass spectra were recorded on a JEOL JMS-T100GCV mass spectrometer. Cyclic voltammetry (CV) was performed using a Bio-Logic VSP-3e instrument. The oxidation potential was measured using glassy carbon as the anode (φ 3 mm), platinum as the cathode (φ 3 mm), and Ag/AgCl as the reference electrode. Merck pre-coated silica gel F_{254} plates (thickness: 0.25 mm) were used for thin-layer chromatography (TLC). All materials were obtained from TCI Fine Chemicals, Wako Pure Chemical Industries, Kanto Chemical, and Sigma-Aldrich and were used without purification. Silica gel column chromatography was performed using Kanto Chemical Silica Gel 60N (spherical, neutral, 63-210 μm).

Abbreviations

BOM, benzyloxymethyl; DBU, 1,8-diazabicyclo[5.4.0]undec-7-ene; DMAP, 4-dimethylaminopyridine; DMF, *N*,*N*-dimethylformamide; HFIP, 1,1,1,3,3,3-hexafluoro-2-propanol; MVK, methyl vinyl ketone; THF, tetrahydrofuran; TIPDS, tetraisopropyldisiloxan-1,3-diyl

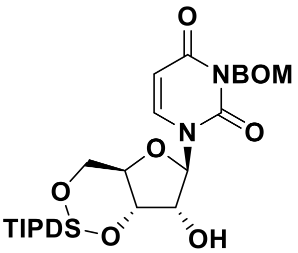

3-((Benzyloxy)methyl)-1-((6aR,8R,9R,9aS)-9-hydroxy-2,2,4,4-tetraisopropyltetrahydro-6H-furo[3,2-f][1,3,5,2,4]trioxadisilocin-8-yl)pyrimidine-2,4(1H,3H)-dione **(S1):** To a solution of uridine (2.44 g, 10 mmol) in pyridine (50 mL) was added $TIPDSCl_2$ (3.28 mL, 10.5 mmol). After stirring at rt for 40 h, MeOH (5 mL) was added to the reaction mixture, and the solvent was removed in vacuo. The resulting residue was partitioned between EtOAc (100 mL) and water (200 mL), and the aqueous layer was extracted with EtOAc (3 × 30 mL). The combined organic layers were dried over anhydrous Na_2SO_4 and the filtrate was concentrated in vacuo. The resulting crude product was dissolved in DMF (50 mL) and DBU (2.99 mL, 20 mmol) and BOM-Cl (2.06 mL, 15 mmol) were added at 0 °C. After stirring for 1 h, MeOH (5 mL) was added to the reaction mixture, which was then partitioned between EtOAc (50 mL) and water (100 mL). The aqueous layer was extracted with EtOAc (3 × 50 mL), and the combined organic layer was washed with water (2 × 200 mL) and brine (200 mL) and dried over anhydrous Na_2SO_4. After the filtrate was concentrated in vacuo, the resulting residue was purified by silica gel column chromatography (Hex/EtOAc = 2:1 to 2:3) to obtain 4.925 g of the title compound as a colorless oil (8.12 mmol, 81% over two steps).

^{1}H NMR (500 MHz, $CDCl_3$) δ 7.63 (d, J = 8.1 Hz, 1H, H-6), 7.39 – 7.29 (m, 4H, Ph), 7.26 (d, J = 0.8 Hz, 1H, Ph, overlapped with $CHCl_3$), 5.73 (s, 1H, H-1'), 5.71 (d, J = 8.2 Hz, 1H, H-5), 5.52 – 5.43 (m, 2H, $NC\underline{H_2}O$), 4.72 (s, 2H, $OC\underline{H_2}Ph$), 4.35 (dd, J = 8.8, 4.8 Hz, 1H, H-2'), 4.20 (d, J = 12.3 Hz, 1H, H-4'), 4.13 (d, J = 4.8 Hz, 1H, H-3'), 4.10

(dt, J = 9.0 Hz, 1H, H-5'a), 4.00 (dd, J = 13.2, 3.0 Hz, 1H, H-5'b), 1.13 – 0.98 (m, 28H, $SiCH(CH_3)_2$ x 4); **^{13}C NMR (126 MHz, CDCl$_3$)** δ 162.68, 150.78, 138.61, 138.08, 128.32, 127.68, 127.65, 101.57, 91.47, 82.01, 75.29, 72.44, 70.41, 69.21, 60.43, 17.48, 17.42, 17.32, 17.30, 17.11, 17.04, 16.99, 16.91, 13.50, 13.01, 12.98, 12.65; **HRMS (ESI)** Calcd. for $[C_{29}H_{47}N_2O_8Si_2]^+$ 607.2861, found 607.2858.

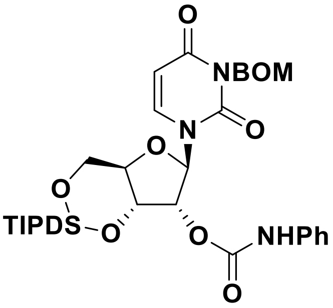

(6aR,8R,9R,9aR)-8-(3-((Benzyloxy)methyl)-2,4-dioxo-3,4-dihydropyrimidin-1(2H)-yl)-2,2,4,4-tetraisopropyltetrahydro-6H-furo[3,2-f][1,3,5,2,4]trioxadisilocin-9-yl phenylcarbamate **(1):** To a solution of **S1** (5.11 g, 8.42 mmol) in THF (42 mL) was added DBU (1.26 mL, 8.42 mmol) and PhNCO (912 µL, 8.42 mmol) and the reaction was stirred at rt for 2 h. the reaction mixture was concentrated in vacuo, and the resulting residue was purified by silica gel column chromatography (Hex/EtOAc = 2:1) to afford 5.32 g of the title compound as a white amorphous solid (7.33 mmol, 87%).

Note: This compound has the same R_f value as **S1**.

^{1}H NMR (400 MHz, CDCl$_3$) δ 7.62 (d, J = 8.2 Hz, 1H, H-6), 7.43 – 7.28 (m, 9H, Ph), 7.08 (t, J = 7.5 Hz, 1H, Ph), 6.76 (s, 1H, NH), 5.86 (s, 1H, H-1'), 5.72 (d, J = 8.2 Hz, 1H, H-5), 5.53 – 5.43 (m, 2H, $NC\underline{H_2}O$), 5.35 (d, J = 5.0 Hz, 1H, H-2'), 4.71 (d, J = 4.0 Hz, 2H, $OC\underline{H_2}Ph$), 4.40 (dd, J = 9.2, 5.0 Hz, 1H, H-3'), 4.22 (d, J = 12.0 Hz, 1H, H-4'), 4.06 – 3.96 (m, 2H, H-5'ab), 1.16 – 0.93 (m, 28H, $SiCH(CH_3)_2$ x 4); **^{13}C NMR (126 MHz, CDCl$_3$)** δ 162.73, 162.57, 150.71, 138.10, 137.73, 129.12, 129.08, 128.38,

127.74, 127.70, 127.67, 123.79, 102.02, 101.62, 89.41, 82.43, 76.27, 72.49, 70.59, 68.22, 59.92, 17.52, 17.47, 17.36, 17.34, 17.15, 17.08, 17.02, 17.00, 16.97, 16.95, 16.93, 16.91, 13.91, 13.61, 13.54, 13.32, 13.12, 13.05, 13.03, 13.02, 13.00, 12.80, 12.71, 12.70; **HRMS (ESI)** Calcd. for $[C_{36}H_{52}N_3O_9Si_2]^+$ 726.3237, found 726.3229.

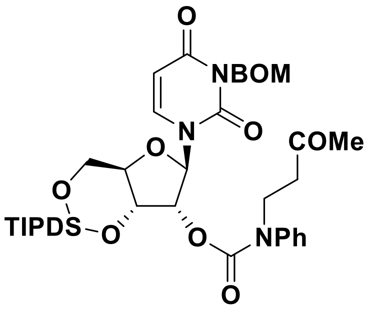

***(6aR,8R,9R,9aR)-8-(3-((Benzyloxy)methyl)-2,4-dioxo-3,4-dihydropyrimidin-1(2H)-yl)-2,2,4,4-tetraisopropyltetrahydro-6H-furo[3,2-f][1,3,5,2,4]trioxadisilocin-9-yl (3-oxobutyl)(phenyl)carbamate* (3):** Compound **1** (145 mg, 0.2 mmol), Bu_4NPF_6 (387 mg, 1 mmol), CH_2Cl_2 (10 mL), phosphate base (90 mg, 0.2 mmol) and methyl vinyl ketone (32.7 μL, 0.4 mmol) were added to a test tube, which was then subjected to a constant electrical current of 5 mA (3 F/mol, 57.9 C) through the CF anode (1 × 1 cm) and the Pt cathode (1 × 1 cm). The reaction mixture was concentrated in vacuo and Et_2O (20 mL) was added. The resulting precipitate was removed by filtration through a short silica gel pad under reduced pressure. The filtrate was concentrated in vacuo and the resulting residue was purified by silica gel column chromatography (Hex/EtOAc = 3:1 to 2:1) afforded 78 mg of the title compound as a colorless oil (0.098 mmol, 49%). The analytical yield was determined based on ^{1}H NMR spectra, using benzaldehyde (20.4 μL, 0.2 mmol) as an internal standard and the integral of $NC\underline{H_2}CH_2COMe$ in **3** was compared with that of the reference peak ($C\underline{H}O$ of benzaldehyde).

^{1}H NMR (500 MHz, $CDCl_3$) δ 7.84 (d, J = 8.1 Hz, 1H, H-6), 7.41 – 7.20 (m, 9H, Ph,

overlapped with $CHCl_3$), 7.07 (m, 1H, Ph), 6.12 (d, J = 3.6 Hz, 1H, H-1'), 5.73 (d, J = 8.2 Hz, 1H, H-5), 5.47 (s, 2H, NC$\underline{H_2}$O), 5.20 – 5.16 (m, 1H, H-2'), 4.68 (s, 2H, OC$\underline{H_2}$Ph), 4.53 (t, J = 5.5 Hz, 1H, H-3'), 4.14 – 4.05 (m, 3H, H-4', H-5'ab), 2.44 (t, 2H, C$\underline{H_2}$COCH$_3$), 2.13 (s, 3H, COC$\underline{H_3}$), 1.56 (q, J = 3.5 Hz, 2H, NC$\underline{H_2}$CH$_2$), 1.14 – 0.92 (m, 28H, SiCH(CH$_3$)$_2$ x 4); **^{13}C NMR (126 MHz, CDCl$_3$)** δ 162.65, 151.28, 138.56, 138.09, 129.27, 128.46, 127.80, 127.78, 124.31, 102.47, 87.83, 84.63, 72.47, 70.62, 69.00, 61.32, 43.56, 30.00, 23.38, 17.49, 17.42, 17.40, 17.35, 13.59, 13.48, 13.15, 13.10, 13.00, 0.13; **HRMS (APCI)** Calcd. for $[C_{40}H_{58}N_3O_{10}Si_2]^+$ 796.3655, found 796.3646.

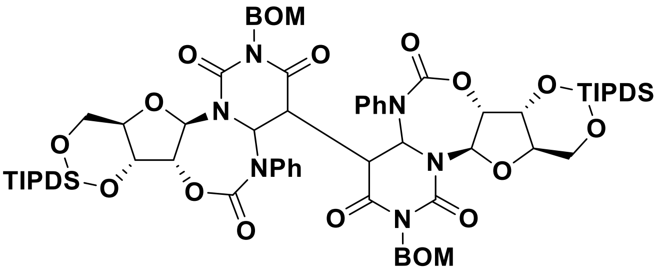

***(7aR,7bR,7'aR,7'bR,13aR,13'aR,14aR,14'aR)-2,2'-Bis((benzyloxy)methyl)-9,9,9',9',11,11,11',11'-octaisopropyl-5,5'-diphenyldodecahydro-1H,1'H,13H,13'H-[4,4'-bis[1,3,5,2,4]trioxadisilocino[6',7':4,5]furo[2,3-f]pyrimido[6,1-d][1,3,5]oxadiazepine]-1,1',3,3',6,6'(2H,2'H,4H,4'H)hexaone* (4):** Compound **1** (145 mg, 0.2 mmol), Bu_4NPF_6 (387 mg, 1 mmol), CH_2Cl_2 (10 mL), phosphate base (90 mg, 0.2 mmol), methyl vinyl ketone (32.7 μL, 0.4 mmol), and HFIP (41.5 μL, 0.4 mmol) were added to a test tube, which was then subjected to a constant electrical current of 5 mA (3 F/mol, 57.9 C) through the CF anode (1 × 1 cm) and the Pt cathode (1 × 1 cm). The reaction mixture was concentrated in vacuo and Et_2O (20 mL) was added. The resulting precipitate was removed by filtration through a short silica gel pad under reduced pressure. The filtrate was concentrated in vacuo and the resulting residue was passed through a short pad of silica gel (Hex/EtOAc = 1:2, containing 0.5% Et_3N) and further purified by preparative

TLC (Hex:EtOAc = 2:3) to afford 39.7 mg of the title compound as a brown oil (0.027 mmol, 27%). The analytical yield was determined based on ^{1}H NMR spectra, using benzaldehyde (20.4 µL, 0.2 mmol) as an internal standard and the integral of SiC<u>H</u>$(CH_3)_2$ in **4** was compared with that of the reference peak (C<u>H</u>O of benzaldehyde). **^{1}H NMR (500 MHz, $CDCl_3$)** δ 7.68 (d, J = 7.9 Hz, 1H, Ph), 7.62 (d, J = 8.2 Hz, 1H, Ph), 7.40 – 7.20 (m, 18H, Ph), 5.83 (s, 1H, H-1'), 5.73 – 5.66 (m, 3H, H-1', NC<u>H</u>$_2$O), 5.52 – 5.37 (m, 6H, NC<u>H</u>$_2$O, H-2' x 2, H-3' x 2), 4.71 (s, 4H, OC<u>H</u>$_2$Ph x 2), 4.46 – 3.86 (m, 10H, H-4' x 2, H-5'ab x 2, H-5 x 2, H-6 x 2), 1.64 (m, 4H, SiC<u>H</u>$(CH_3)_2$ x 4, overlapped with H_2O), 1.35 (m, 4H, SiC<u>H</u>$(CH_3)_2$ x 4), 1.16 – 0.85 (m, 48H, SiCH(C<u>H</u>$_3)_2$ x 8); **^{13}C NMR (126 MHz, $CDCl_3$)** δ 162.67, 151.85, 150.69, 138.17, 130.24, 129.16, 129.04, 128.57, 128.44, 128.43, 127.75, 127.73, 101.91, 89.39, 89.17, 82.28, 82.06, 76.61, 72.54, 70.62, 69.28, 69.19, 68.20, 68.03, 59.61, 32.32, 32.30, 32.25, 23.38, 18.68, 18.67, 17.57, 17.52, 17.51, 17.41, 17.39, 17.37, 17.21, 17.08, 17.04, 17.00, 16.98, 13.69, 13.64, 13.62, 13.08, 12.81, 12.76; **HRMS (APCI)** Calcd. for $[C_{72}H_{101}N_6O_{18}Si_4]^+$ 1449.6244, found 1449.6250.

II. Simulation for cyclic voltammogram (Figures S1 and S2)

CV simulation was performed using DigiElch 8 (Gamry Instruments).

Simulation parameters:

1 $\rightleftharpoons$ **1**$^{\bullet+}$ + e$^-$ $E^0 = 2.0$ V; $k_{s(\mathbf{1})} = 1$ cm s^{-1}

1 + phosphate base $\rightleftharpoons$ **1-phosphate base** + e$^-$ $E^0 = 1.8$ V; $k_{s(\mathbf{1}\text{+phosphate base})} = 0.1$ cm s^{-1}

1 + phosphate base + HFIP $\rightleftharpoons$ **1-phosphate base-HFIP** + e$^-$ $E^0 = 1.8$ V; $k_{s(\mathbf{1}\text{+phosphate base+HFIP})} = 0.01$ cm s^{-1}

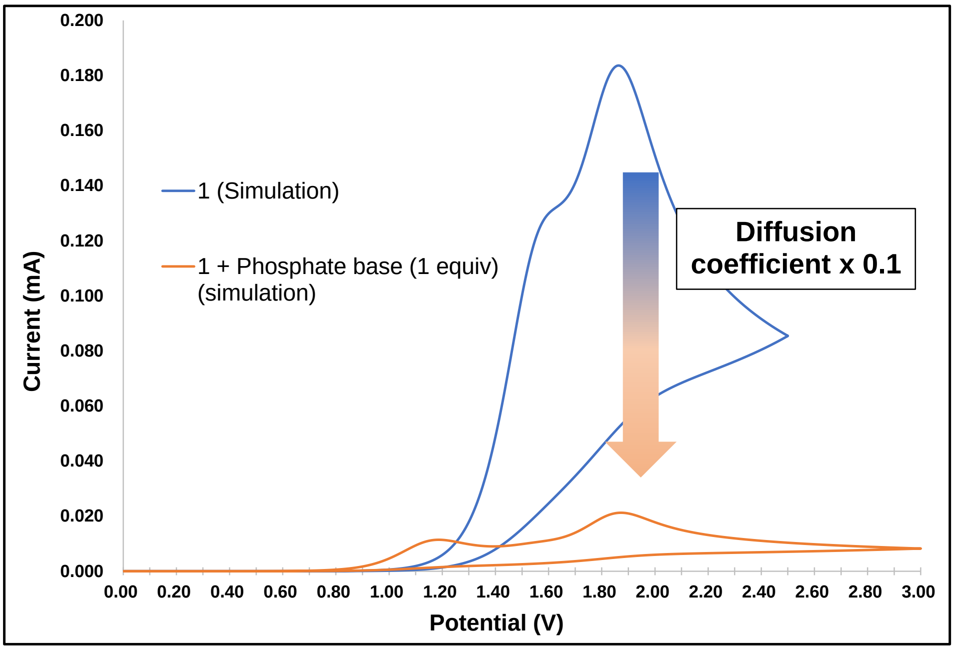

Figure S1. CV simulation for **1** and **1-phosphate base** complex.

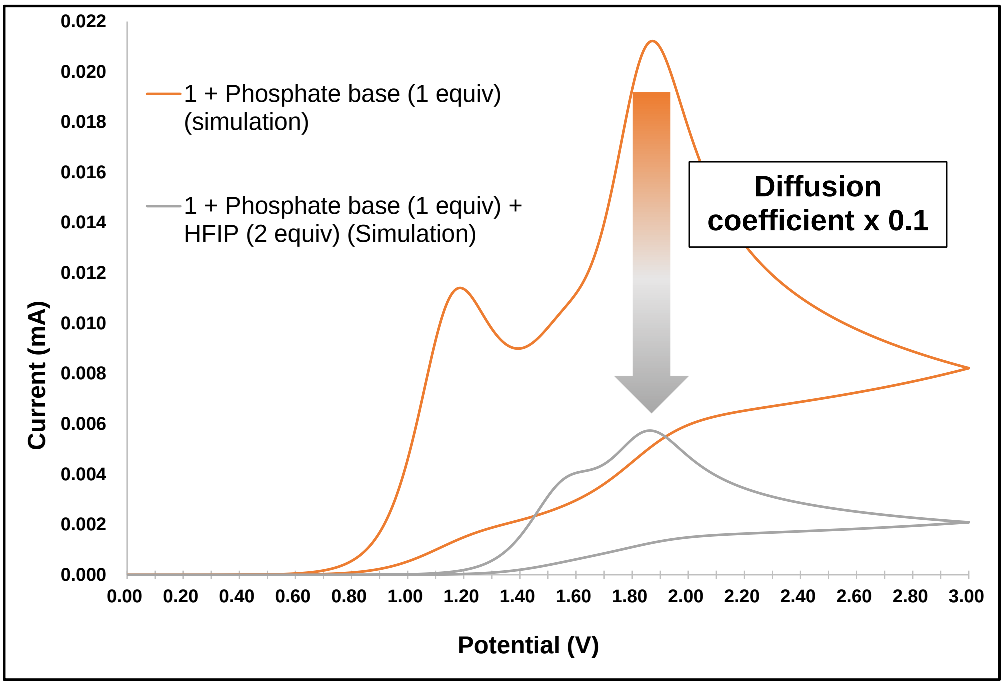

Figure S2. CV simulation for **1-phosphate base-HFIP** complex.

We hypothesized that the two- or three-component hydrogen bond complexes (**1-phosphate base** and **1-phosphate base-HFIP**) would have a diffusion coefficient smaller than that of **1**. Although a decrease in the diffusion coefficient decreased the current value, similar to our experimental results, the reported diffusion coefficient of the hydrogen-bond complex between the amide and phosphate base is only twice as small as that of the sole amide molecule (Gschwind et al., *J. Am. Chem. Soc.*, **2021**, *143*, 724-735.), while our simulated diffusion coefficient was unrealistically smaller than previously reported values. Therefore, we concluded that the diffusion coefficient is not a major factor affecting the CV behavior in the present study.

III. ^{1}H and ^{13}C NMR spectra of compounds

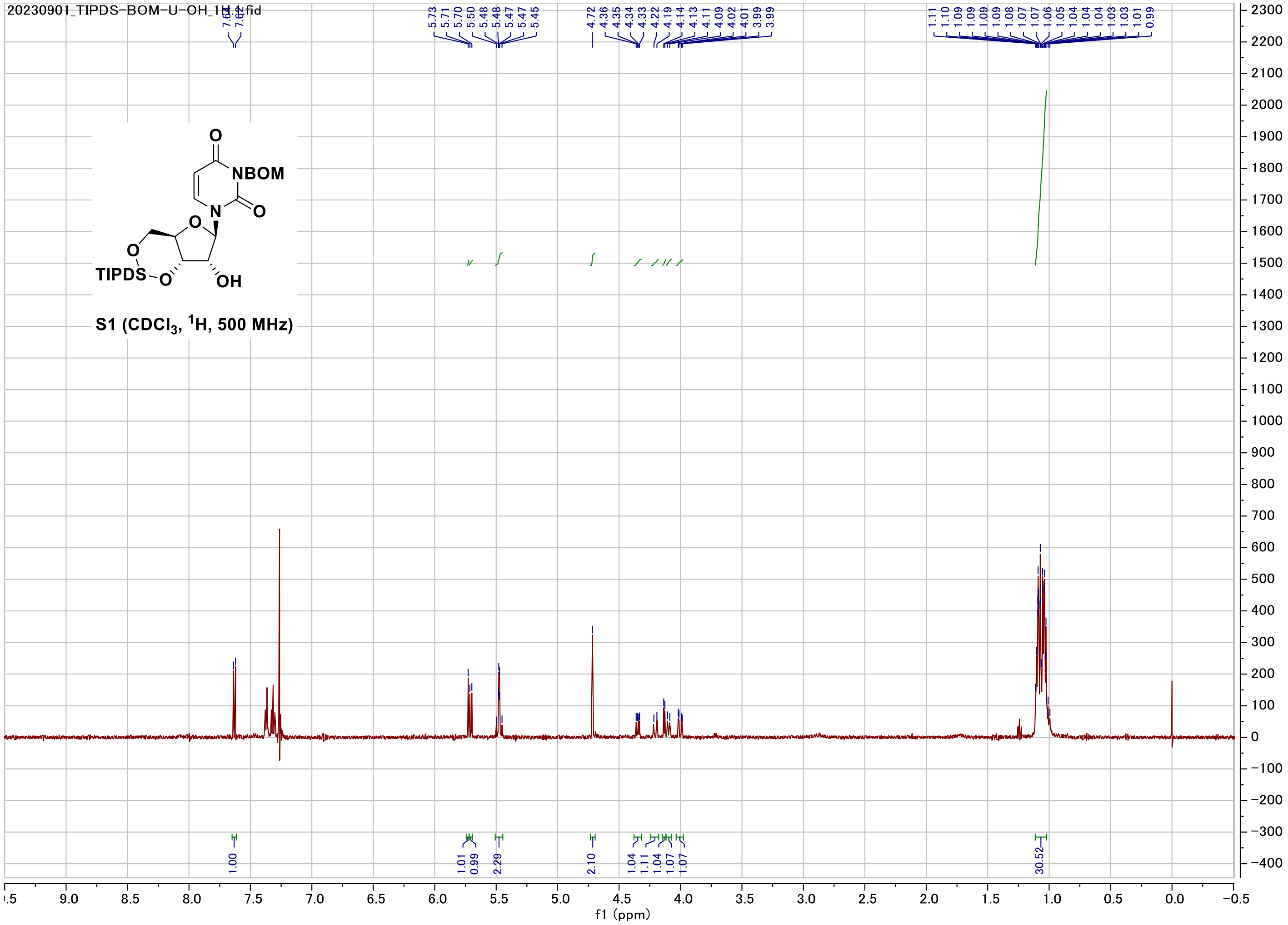

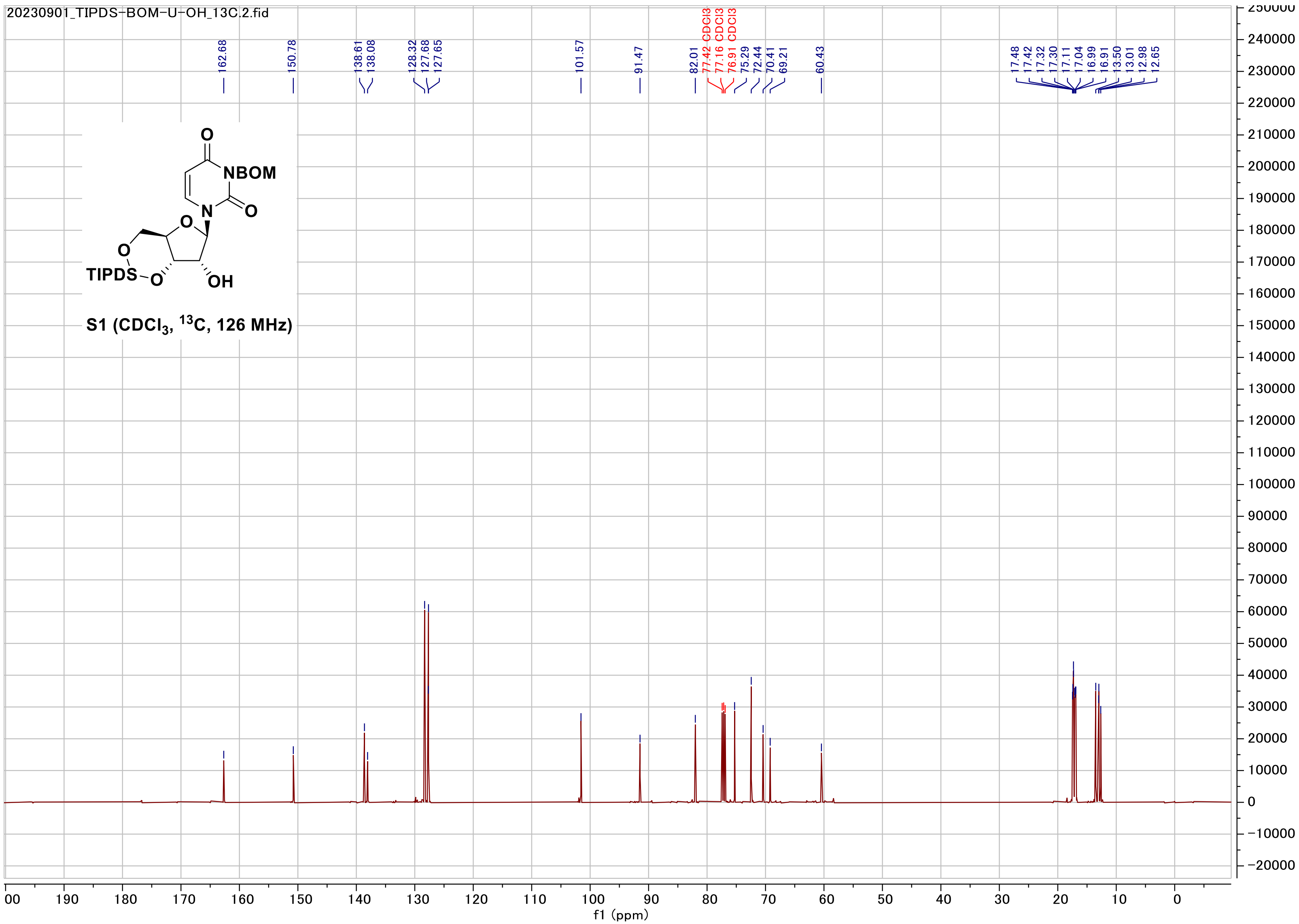
20230901_TIPDS-BOM-U-OH_13C.2.fid
162.68
150.78
138.61
138.08
128.32
127.68
127.65
101.57
91.47
82.01
77.42 CDCl3
77.16 CDCl3
76.91 CDCl3
75.29
72.44
70.41
69.21
60.43
17.48
17.42
17.32
17.30
17.11
17.04
16.99
16.91
13.50
13.01
12.98
12.65
O
NBOM
N
O
O
O
TIPDS
O
OH
S1 (CDCl3, 13C, 126 MHz)
f1 (ppm)

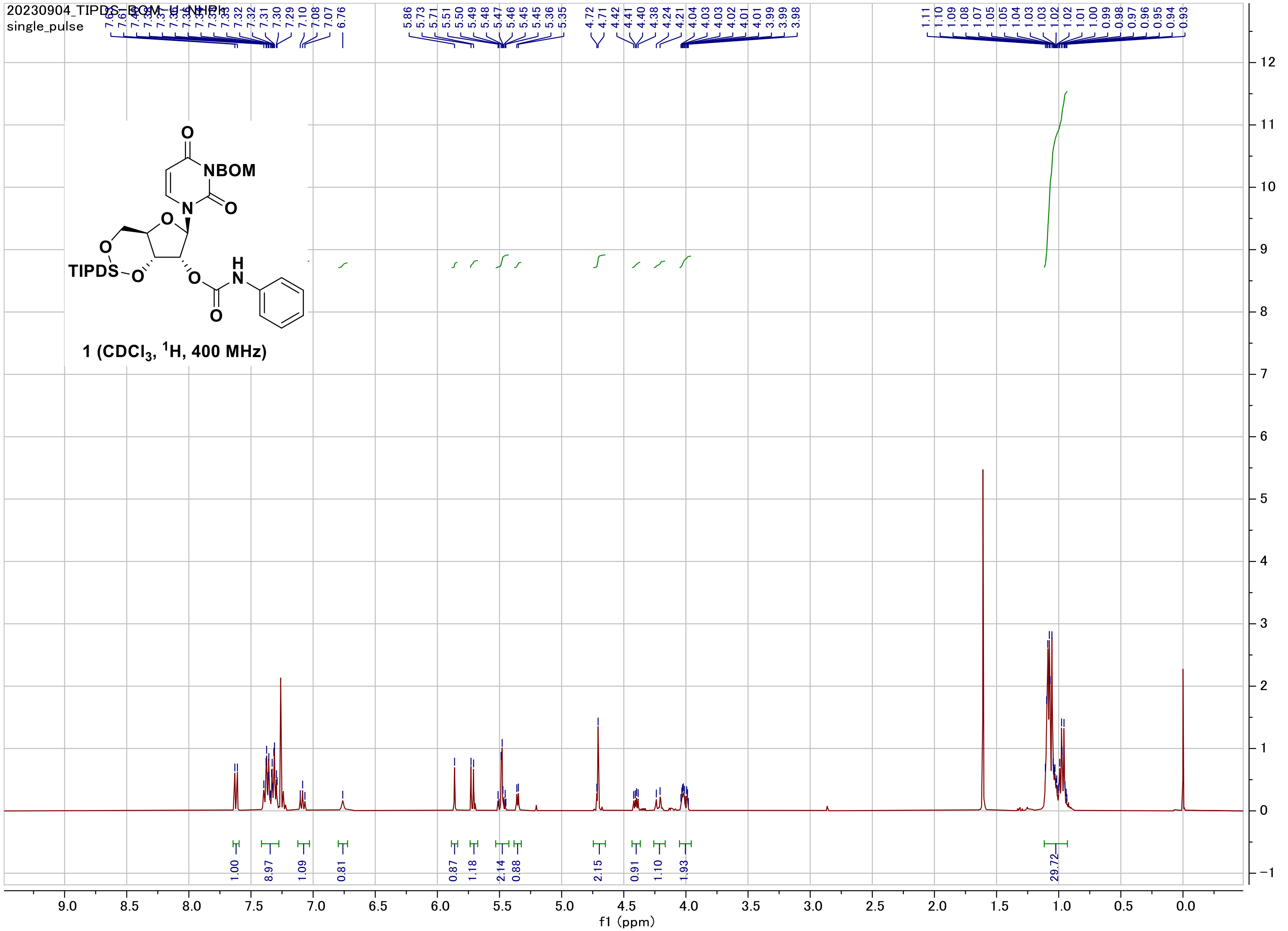
20230904_TIPDS-BOM-U-NHPh
single_pulse
7.31 7.30 7.29 7.10 7.08 7.07 6.76
5.86 5.73 5.71 5.51 5.50 5.49 5.48 5.47 5.46 5.45 5.36 5.35
4.72 4.71 4.42 4.41 4.40 4.38 4.24 4.21 4.04 4.03 4.03 4.02 4.01 4.01 3.99 3.99 3.98
1.11 1.10 1.09 1.08 1.07 1.05 1.05 1.04 1.03 1.03 1.02 1.02 1.01 1.00 0.99 0.98 0.97 0.96 0.95 0.94 0.93
O
NBOM
N
O
O
O
TIPDS
O
O
H
N
O
1 (CDCl3, 1H, 400 MHz)
1.00 8.97 1.09 0.81 0.87 1.18 2.14 0.88 2.15 0.91 1.10 1.93 29.72
9.0 8.5 8.0 7.5 7.0 6.5 6.0 5.5 5.0 4.5 4.0 3.5 3.0 2.5 2.0 1.5 1.0 0.5 0.0
f1 (ppm)
12 11 10 9 8 7 6 5 4 3 2 1 0 −1

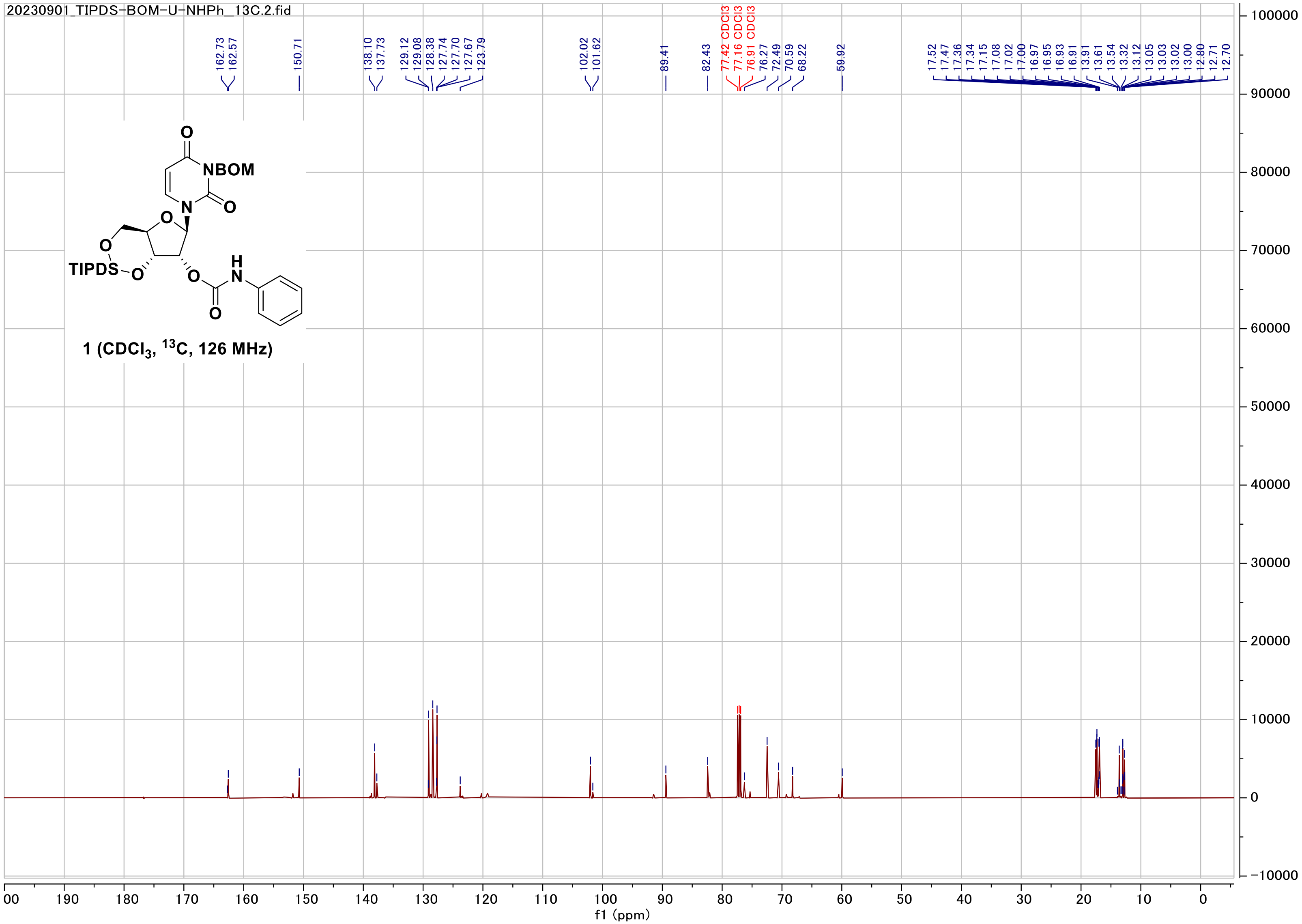
20230901_TIPDS-BOM-U-NHPh_13C.2.fid
162.73
162.57
150.71
138.10
137.73
129.12
129.08
128.38
127.74
127.70
127.67
123.79
102.02
101.62
89.41
82.43
77.42 CDCl3
77.16 CDCl3
76.91 CDCl3
76.27
72.49
70.59
68.22
59.92
17.52
17.47
17.36
17.34
17.15
17.08
17.02
17.00
16.97
16.95
16.93
16.91
13.91
13.61
13.54
13.32
13.12
13.05
13.03
13.02
13.00
12.80
12.71
12.70
NBOM
TIPDS
1 ($CDCl_3$, ^{13}C, 126 MHz)
f1 (ppm)

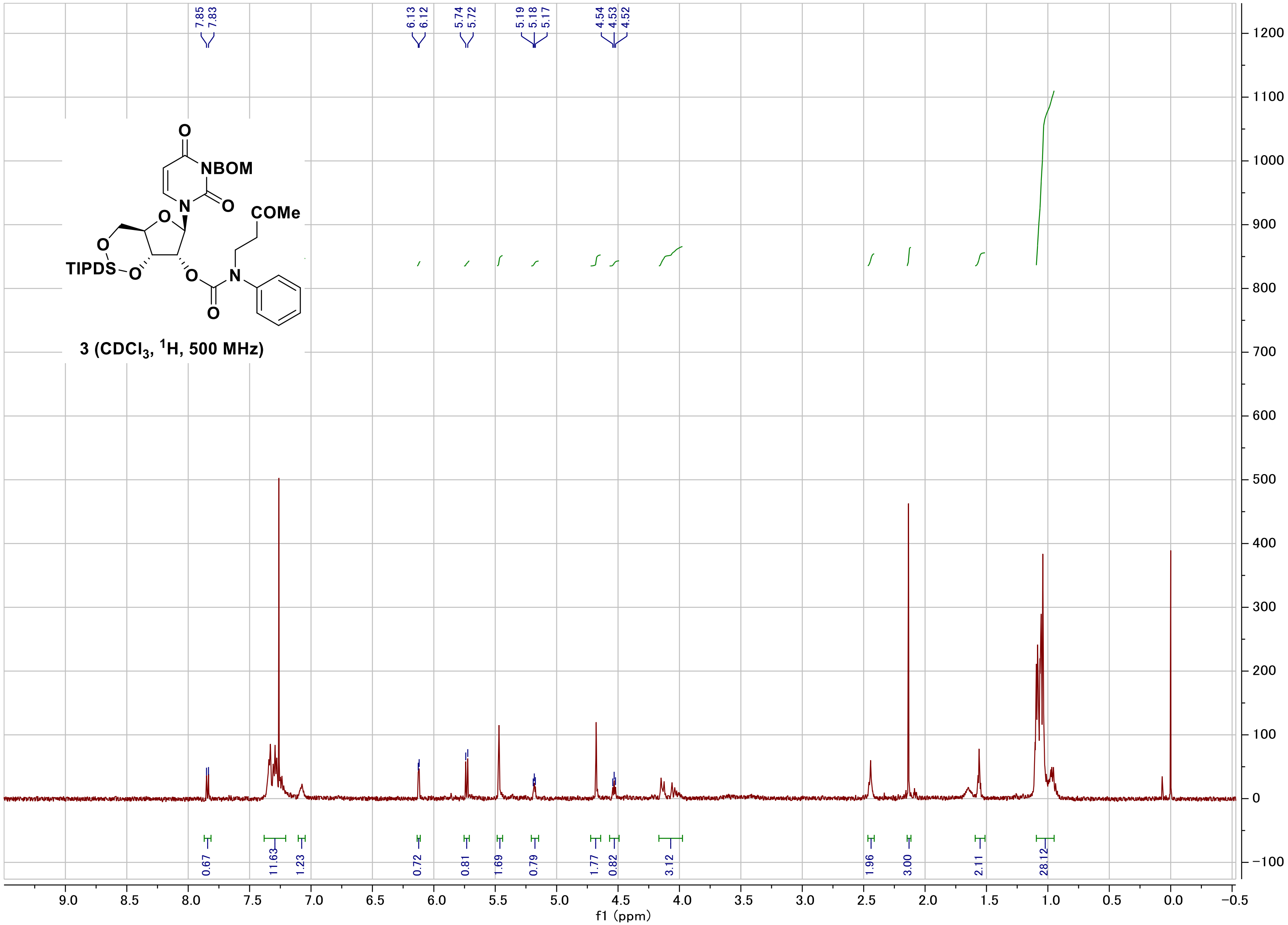
O
NBOM
N
O
COMe
O
O
TIPDS
O
O
N
O
3 (CDCl3, 1H, 500 MHz)
7.85
7.83
6.13
6.12
5.74
5.72
5.19
5.18
5.17
4.54
4.53
4.52
0.67
11.63
1.23
0.72
0.81
1.69
0.79
1.77
0.82
3.12
1.96
3.00
2.11
28.12
f1 (ppm)

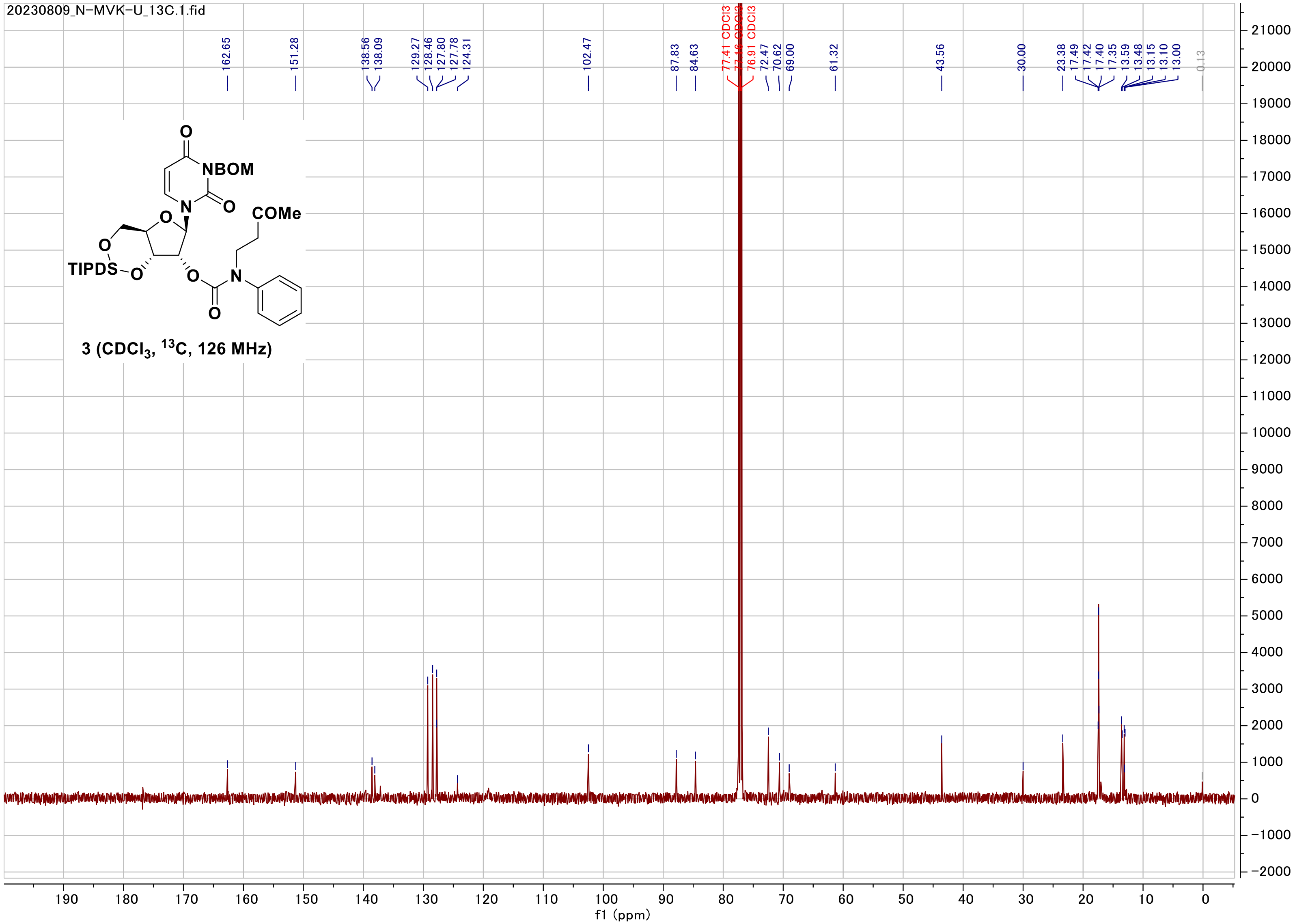

20230809_N-MVK-U_13C.1.fid
162.65
151.28
138.56
138.09
129.27
128.46
127.80
127.78
124.31
102.47
87.83
84.63
77.41 CDCl3
76.91 CDCl3
72.47
70.62
69.00
61.32
43.56
30.00
23.38
17.49
17.42
17.40
17.35
13.59
13.48
13.15
13.10
13.00
0.13
O
NBOM
N
O
O
COMe
O
TIPDS
O
O
N
O
3 (CDCl$_3$, ^{13}C, 126 MHz)
f1 (ppm)

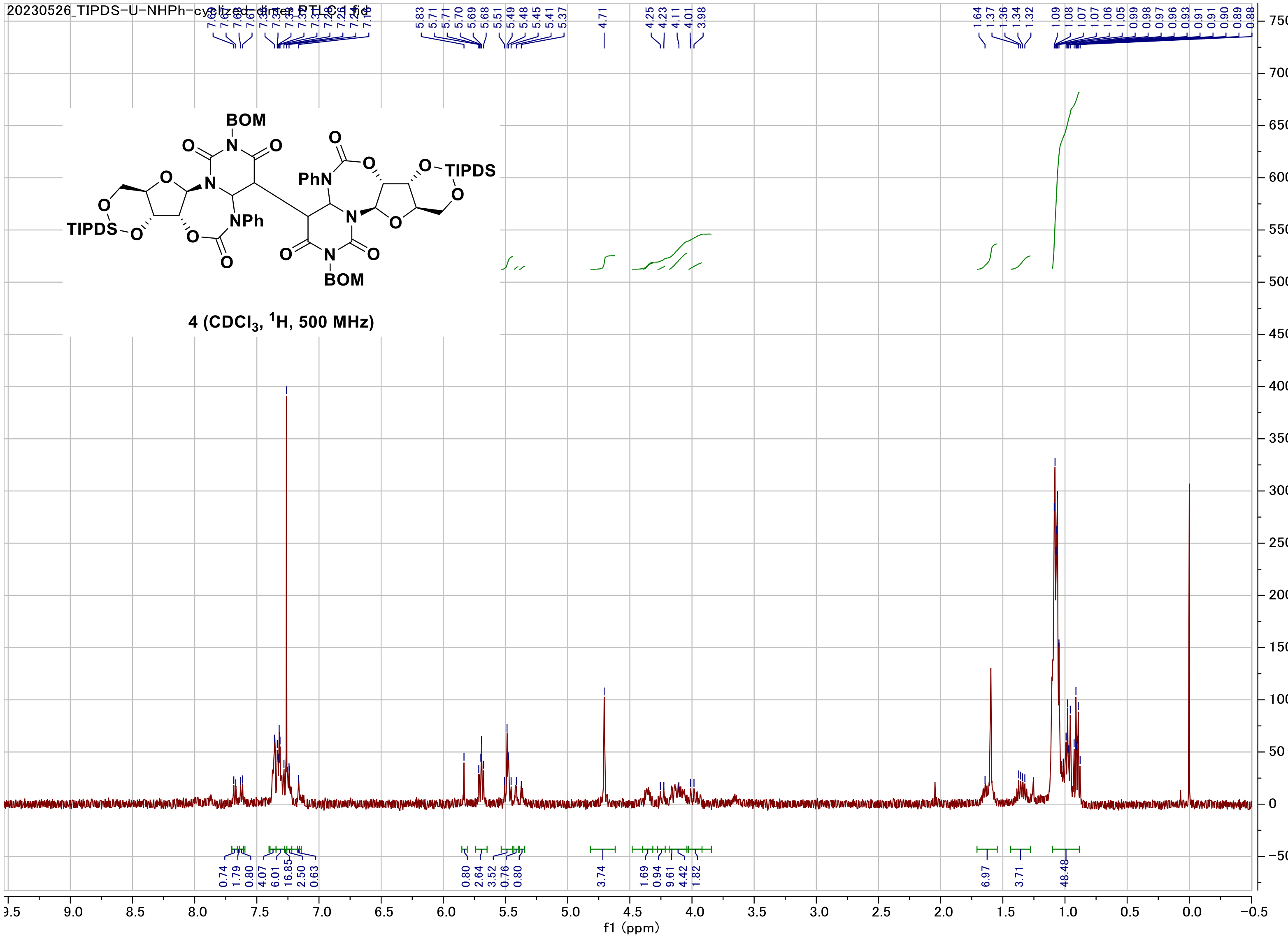

20230526_TIPDS-U-NHPh-cyclized_dimer
4 (CDCl$_3$, ^{1}H, 500 MHz)
f1 (ppm)

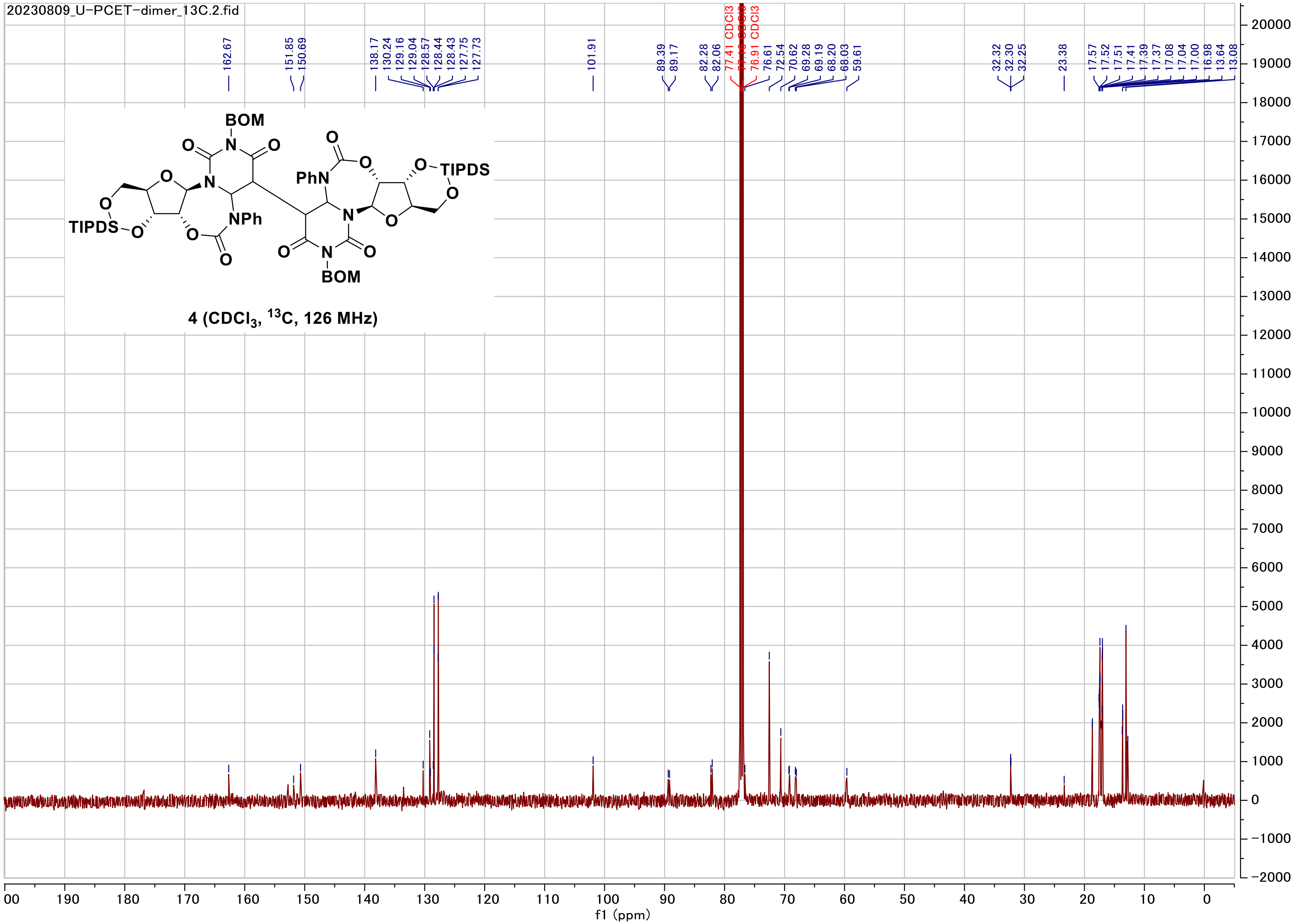

20230809_U-PCET-dimer_13C.2.fid
4 (CDCl3, 13C, 126 MHz)
BOM
TIPDS
NPh
PhN
f1 (ppm)
